# Views of breast cancer survivors on work participation guidance by general practitioners: a qualitative study

**DOI:** 10.1186/s12875-022-01768-x

**Published:** 2022-06-17

**Authors:** Marie-Christine Sarfo, Kristel M. van Asselt, Monique H. Frings-Dresen, Femke de Jong, Nynke van Dijk, Angela G. E. M. de Boer

**Affiliations:** 1 Amsterdam UMC, Department General Practice, Location University of Amsterdam, Amsterdam Public Health Research Institute, Amsterdam, Netherlands; 2 Amsterdam UMC, Department Public and Occupational Health/Coronel Institute of Occupational Health, Location University of Amsterdam, Amsterdam Public Health Research Institute, Amsterdam, Netherlands

**Keywords:** Focus groups, General practice, Qualitative research, Return to work, Cancer survivors

## Abstract

**Background:**

Breast cancer survivors can be at high risk of having work-related problems. Previous studies suggest that GPs could discuss work participation with cancer patients and provide guidance.

The aim this study is to explore the experiences and expectations of breast cancer survivors with their GPs’ role regarding guidance on work participation and return to work.

**Methods:**

A qualitative study with Dutch female breast cancer survivors was held in the Netherlands. Four focus groups with 25 participants were conducted and audio-taped. Transcripts were analysed using thematic analysis.

**Results:**

Breast cancer survivors reported a wide range of experiences with guidance from their GPs regarding work participation. Patients who contacted their GPs with work-related issues felt listened to during the consultation. Some patients experienced very limited or no guidance, while others were intensively guided by their GPs. The guidance was provided in the form of counseling, psychosocial support, and referral to other health care professionals. When cancer patients experienced problems with reintegration in work, they expected GPs to have a supportive and referring role in work participation guidance.

**Conclusion:**

In returning to work, breast cancer survivors expect their GPs to play a supportive role, especially when they encounter difficulties in reintegrating. However, their actual experience of guidance received from their GPs varied, from none received at all, to intensive support provided.

## Key message

- Breast cancer survivors who contact their GPs with work-related issues feel listened to during the consultation
- GPs can discuss work participation with cancer survivors and provide guidance
- Breast cancer survivors experienced supportive, limited or referral roles in work guidance by general practitioners
- Survivors with work problems expect support and referral from general practitioners

## Background

Breast cancer is the most common type of cancer among women worldwide [1]. The survival rate of breast cancer patients has increased significantly in the last decades, due to advances in early detection through screening and improvements in diagnosis and treatment [2, 3]. In cancer survivorship there has been an increasing focus on work participation and return to work (RTW) [4]. In breast cancer survivors (BCSs), work is an important and meaningful activity [5]. Work participation can have a positive influence on survivors’ health and well-being [6]. Moreover, RTW is a part of their social recovery and contributes to the quality of life [7, 8].

Most BCSs can continue or return to work, but multiple factors affect their workability [9–14]. Chemotherapy, extensive surgery, disease-related fatigue, and exhaustion can constrain BCSs in RTW, as well as job stress and lack of colleague and employer support [7, 9, 15, 16].

In the Netherlands, GPs can refer employed BCSs to an occupational physician who is an expert at helping employees maintain or regain workability, however, not all breast cancer patients visit one after diagnosis and treatment [17]. Other BCSs don´t have access to an occupational physician [18, 19]. However, from the very beginning of the trajectory, general practitioners (GPs) are an important access point in health care for BCSs. During diagnosis, treatment, and aftercare, medical information about the cancer patient is exchanged between involved specialists and GPs [20]. GPs can have an important role in discussing work and providing guidance [21]. According to The Dutch College of General Practitioners (NHG), GPs need to focus on the somatic, psychosocial aspects and (return to) work of cancer patients during the cancer treatment phase [22]. However, it is unknown how BCSs experience this guidance by GPs. Therefore, this study aims to define the GP’s role in providing work participation and RTW guidance to BCSs, from the perspective of the BCS. The objectives were to assess: 1) BCSs’ experiences of their GPs’ role in work participation guidance and RTW, and 2) BCSs expectations from their GPs regarding work participation guidance and RTW.

## Methods

The reporting of the methods and results of this qualitative study were based on the Consolidated Criteria for Reporting Qualitative Studies checklist (COREQ) [23]. Ethical approval was waived by the Medical Ethical Committee of the Amsterdam UMC, as The Medical Research Involving Human Subjects Act (WMO) does not apply to this study (W16_140 # 16.163).

### Participants

Participants were recruited through The Dutch Breast Cancer Association (BVN), a professional patient organization for and by individuals with breast cancer, consisting of volunteers.

BVN sent an online questionnaire to all their BCSs members about their experiences with psychosocial guidance from their GPs in RTW. The respondents were invited to enter their email address when interested in a focus group discussion on ‘psychosocial guidance from the GP’ with researchers from our department. All participants provided written informed consent before participation. Between May 31 and June 3, 2016, 598 respondents with paid employment prior to breast cancer diagnosis completed the questionnaire. One hundred and six respondents were interested in participating in our study. Through email, these 106 respondents were subsequently sent information about the study and invited to participate. Reasons for non-participation could not be collected due to privacy regulations.

### Setting and data collection

In August 2016, four focus groups were conducted by one experienced moderator NvD (MD., Professor General Practice), and a trained observer FdJ (MD., junior researcher/ GP in training), at various facilities. No relationships between researcher and patients existed prior to the focus groups.

A short questionnaire was completed by the participants to assess demographics, including age, marital status, level of education, and working hours per week.

During the focus groups, the moderator used a topic guide for structured guidance. The role of the GP regarding breast cancer and work/re-integration during this period was discussed. The topic guide was based on the literature [4–22] and the experiences of the researchers. Questions mentioned in the topic guide are summarized in Table 1.

**Table 1:** Questions in the topic guide

Do you need support from your GP to return to work?;
What does that support require?;
What is your experience: Who did you consult?;
Should the GP ask about your work, or only discuss it when it is important to you (so if you ask yourself)?;
At what moment should the GP talk about it?

The focus groups were audio-recorded and field notes were made by FdJ. The mean duration of the four focus groups was 80 min.

### Data analysis

The recordings from the focus groups were transcribed verbatim. All respondents’ data were anonymized. Transcripts were not returned to the respondents. Two members of the research team (FdJ and MCS, both GPs in training/ junior researchers), separately checked the audio recordings with the transcripts. The transcripts were read at least two times by the two researchers (FdJ, MCS) and initial ideas were noted.

The qualitative data analysis software, MAXQDA 2020 was used to analyze the data. [24] An inductive approach was used to generate codes and themes from the qualitative data.

[25] Three members (FdJ, MCS and KvA) of the research team separately coded the transcript of the first focus group. The initial codes of the three members were compared and discussed. The transcripts of the other three focus groups were independently coded by one member (MCS), codes were added to the coding tree. The meaning of the codes were refined and, if deemed necessary, re-coding was performed in discussion with the research team (MCS, KvA, MHFD, NvD, and AdB, all females).

The different codes were grouped into themes and subthemes by MCS, in close consultation with senior researchers AdB and KvA. Themes and subthemes were discussed with the research team until consensus was achieved. Data saturation was reached after three focus groups as no new themes and sub-themes emerged when analyzing the fourth focus group.

## Results

Sixty-one women agreed to participate and received additional information about the date and place of the focus groups. Thirty-six of the 61 eligible participants were unable to attend for various reasons, such as physical limitations, traveling distance to the focus group locations, and other planned activities on the data of the focus groups. Therefore, in total, twenty-five BCSs participated in our study in four focus groups (*n* = 9, *n* = 5, *n* = 4, *n* = 7).

The characteristics of the BCSs are summarized in Table 2. The twenty-five participants were diagnosed between 1999 and 2015, the median time since diagnosis was 51 months. The participants were all women between the ages of 39 and 64 years and all Dutch-speaking. Twenty-three out of the 25 participants were of Dutch nationality. The majority of the women had a high level of education (68%) and were working part-time (52%).

**Table 2:** Demographics

Variable	Breast cancer survivors (*N* = 25)	(%)
**Age**, mean in years, (range; standard deviation)	51 (31–64; 7.3)	
**Gender, female**	25	100
**Marital status**		
Married	11	44
Living with partner	5	20
Single	9	36
**Level of education**		
Low	8	32
High	17	68
**Working hours (per week)**		
Not working		
Part-time	5	20
Full-time	13	52
Missing	5 2	20 8
**Time since diagnosis median** in months, (range; standard deviation)	51 (10 – 214; 59)	

Two main themes emerged from the data: ‘experiences of BCSs with their GP’s role in work guidance’ and ‘expectations of BCSs for their GP’s role in work guidance’. The two main themes were organized into the sub-themes namely ‘advice and supportive role’, ‘referral role’, and ‘limited or no role’.

## Experiences of breast cancer survivors with their general practitioners’ role in work guidance

Most patients who contacted their GPs with work-related issues felt listened to during the consultation. In these consultations, the GPs had advising, supportive, and referring roles.

### Advice and supportive role

Patients received advice from GPs on gradually reintegrating into work and suggestions to solve work-related problems. Some patients experienced this as helpful, while others felt it was inappropriate or that the GPs could not help them. One patient said:

‘The GP outlined that it is really not advisable to do full time work. I needed to slow down and also got similar advice from others. So from the GP who knows me well, as well as the occupational health doctor who doesn’t know me well’. (P18)

On the contrary one breast cancer survivor explained:

‘I used to have a GP downtown, when I was at risk of losing my job, he said, “What do you care, take your pocket money home.” At that moment, I thought, what kind of reaction is that?’ (P1)

Another breast cancer patient said:

‘In my case, the GP guidance was minimal during reintegration. I talked to him a few times. The whole reintegration process was difficult. I worked as a social worker for people with a mental disability. I had numerous problems returning after illness. During that time, I went to the GP a few times. He repeatedly invited me to talk. I had the feeling that I could tell him my whole story, to no avail. He really couldn’t do anything, so that was a pity’. (P24)

The BCSs who received intensive support in the RTW process from their GPs were offered help and invited to discuss work and reintegration problems. A breast cancer survivor explained:

‘When it does not go well with the occupational health doctor or if I experience a little too much pressure to start working again, she (the GP) said she wants to get involved’. (P17)

### Referral role

Furthermore, some patients were referred by their GPs for their RTW problems to other health care professionals, such as occupational health physicians, or a psychologist for more intensive psychosocial support. In some cases, referral to other health professionals was directly requested by the BCS. One patient said:

‘It did not go well with the reintegration, therefore I went to the GP. He referred me to a psychologist and the occupational health physician. The GP did not do much himself, but listened’. (P10)

### Limited or no role

In general, however, the participants did not receive any RTW guidance from the GP, as most responded ‘no’ to the focus group question about it. Also, most BCSs were unsatisfied with this shortcoming. One of the participants said:

‘I returned to work, unfortunately, I got pushed out and I find that very upsetting and my GP was not of any use to me, I am sorry’. (P1)

Another participant explained:

‘Yes, I was able to tell my story, but he (the GP) couldn’t be of any use. He told me that it had to be arranged through the occupational health physician’. (P24)

Some participants indicated that they did not receive but also did not need guidance from their GP because the guidance they received at work was sufficient. They stated that if the situation called for it, they would want to be guided by their GP. One of the breast cancer survivors explained:

‘I was not guided by my GP. He asked me if I needed guidance. I thought that the guidance I received at work was enough’. (P16)

## Expectations of breast cancer survivors for their general practitioners' role in work guidance

### Supportive role

When facing work reintegration problems, the BCSs indicated they expected guidance and support from their GPs. One patient said: ‘If I had problems going back to work, it would be nice to discuss it with the GP’. (P16).

Another patient add: ‘If you are unable to resolve the issue with the occupational health physician, it would always be pleasant if the GP guides you’. (P17).

### Referral role

GPs could contribute suggestions for reintegration and refer to other health professionals. One patient suggested:

‘The GP can mention that he knows people that can help the patient. At first a coach and if it gets more intense, a psychologist, practice assistant perhaps and people who have been through the same experience’. (P22).

### Limited role

If patients were not employed, BCSs thought that the GP's role was limited to suggestions and referrals to organizations that could help unemployed patients with applying for paid or voluntary work. One breast cancer survivor said:

‘GP helping with work, it depends, but in my case, I don’t expect him to find a new job for me. I think that a GP should remain in his field of profession. You can’t overload the GP with everything’. (P5)

Some patients had negative experiences with their occupational health physicians and expected support from their GPs, such as help in resolving disagreements between them and their occupational health physicians.

BCSs also expected GPs to directly communicate with occupational health physicians or even employers to explain their medical and psychosocial situation in relationship to work, as GPs have the best view of both for the patient. One patient said:

‘I don’t know if it’s possible if the GP could have contacted my work and explain things. That could have been nice’. (P24)

The BCSs expected their GPs to be informed about their medical and psychosocial status, especially when communicating with the occupational health physicians about patients’ work-related problems. One participant said:

‘The GP can consult the occupational health physician, but then he needs to know you a little bit. Yes, the GP needs to know you, otherwise, he can’t be of any use’. (P21)

Some BCSs stated that they expected GPs to also document the professions of patients and be informed about the type of work the patient did, their working hours, and the importance of employment for the finances of their household. One of the breast cancer survivors shared:

‘I want the GPs to write down the kind of work the patient does. I want them to know that I’m not a woman who only stays at home, I don’t have a man that looks after the children. I’m alone with my work. I want him to know that I work in shifts’. (P14)

## Discussion

### Summary

Regarding work participation guidance and RTW, BCSs had several experiences with their GPs’ roles—limited or none, advising and supportive, and or a referring capacity. The BCSs expected a supportive, referral, or limited role from their GPs when experiencing work-related issues.

Moreover, BCSs said it is important that GPs are informed about the patient’s medical status, home situation, and profession when guiding work participation and RTW.

### Comparison with existing literature

In our study, some BCSs received advice and support from their GPs regarding work participation. Similar work guidance experiences were found in a cohort study by Söderman et al*.* [26]. In this study, eighty percent of the BCSs had meetings with health care professionals regarding work. Women received advice and support regarding work or were encouraged to work, which led to fewer sickness-related absence days. Those who were encouraged to take sickness absence had more sickness absence days.

The role some BCSs in our study expected from their GPs corresponds with the health professionals’ role in the study of Yagil et al*.* [27]. They reported the perspectives of physicians and nurses on their role in RTW. The health care professionals said that their role was to provide cancer survivors with information and suggestions about RTW.

Furthermore, BCSs in our study expected GPs to be informed of their medical situation, suggesting that correspondence between oncology specialists and the GP is critical. In an Austrian cross-sectional study by Spiegel et al., cancer patients indicated that the information exchange between hospital-based specialists and their GPs is extremely valuable [28].

### Strengths and limitations

To our knowledge, this is a unique study that describes both the experiences and expectations of BCSs with their GPs role regarding work guidance. Another strength of the present study is the in-depth exploration of BCSs experiences and expectations with their GPs guidance.

Our study also had some limitations. One limitation was the selection of the participants. The participants were only recruited through The Dutch Breast Cancer Association (BVN), and their ethnicity and their relatively high level of education (68%) were not taken into account. In practice, patients with a lower level of education are more at risk to have work-related issues, problems with reintegration and finding jobs after dropping out of the workforce [7]. So, it could be that inclusion for other participants had emerged to other themes during the focus group discussions. A further limitation regarding the participants is that we lack clinical and professional data to better describe the patients. A second limitation is that our study took place in 2016, before the COVID-19 pandemic. GPs might have discussed more intensely the impact of return to work of vulnerable cancer patients during the pandemic which could have impacted the experience of patients. No other large changes have taken place in legislation or otherwise, and therefore we do not expect any changes that would have impacted our data.

The time between cancer diagnosis of the participants and the date of the focus group varied between the participants (range 10 – 214 months, median 51 months), this could have led to recall bias of participants. Therefore, some experiences of the BCSs with their GPs’ role regarding work guidance might have been missed in the focus group discussion.

### Implications for research and practice

The diversity of BCSs experiences reflect that the role of the GPs in work participation guidance and RTW has not been well-defined. Outcomes also depend on the personal and work-situation of the BCS and the role of other health professionals. Our results suggest that the GPs advice on work participation and RTW are essential for breast cancer patients and that this could entail multiple roles for GPs.

There are limited work participation and RTW guidelines available for GPs. The latest breast cancer guideline of the Dutch College of General Practitioners contains limited information about RTW after breast cancer.

More extensive cancer and work guidance tools or guidelines need to be developed and implemented to guide GPs in supporting BCSs in this area. However, it is also important to examine the GPs’ experiences, since they may be unfamiliar or lack sufficient training in work guidance and RTW [29].

Bains et al*.* investigated health provider’s work-related guidance for colorectal cancer patients [30]. In this study the work-related information given to patients by providers was conflicting, limited and not systematic. Future research on GP’s view regarding work guidance in breast cancer patients could also be of value, to identify the differences with BCS’s view on the subject.

In the study of Kock et al*.*, GPs consider attention to work-related problems important [31]. The GPs were willing to address the work-related problems and counsel about sick leave but needed better cooperation with occupational physicians and training to develop a more proactive approach to work-related problems. Knowing this, it is possible that GPs need to be introduced to better cancer and work guidelines and gain sufficient training, to provide the work guidance BCSs require.

In a recent study in France by Begue et al*.* it was shown that while the level of knowledge and use of the pre RTW medical consultation by GPs is good, it is not optimal. This could be improved by organising training courses for GPs [32]. This would be an opportunity for GPs in other countries as well.

Additional research is needed to explore both the experiences of GPs with work guidance in breast cancer patients and their view on their professional role in work participation guidance and RTW.

## Conclusion

BCSs expected and experienced diverse work guidance and RTW from their GPs. In the case of work-related issues, breast cancer patients expected work guidance from their GPs, such as advice, support and referral. By offering BCSs the appropriate guidance in work participation and RTW, GPs can contribute to the social recovery of breast cancer patients.

## Data Availability

The datasets used and/or analysed during the current study are not publicly available or available from the corresponding author on reasonable request because of the qualitative nature of the interview data and the impossibility to de-identify the data.
